# HLA-DRB1*01 predicts treatment outcome in juvenile idiopathic arthritis: A retrospective–prospective cohort study

**DOI:** 10.17305/bb.2024.11043

**Published:** 2024-10-31

**Authors:** Adisa Čengić, Sniježana Hasanbegović, Izeta Hamza, Tarik Suljic, Velma Selmanović, Aida Ðozo, Elma Fejzić, Lamija Zečević-Pašić, Nejra Džananović

**Affiliations:** 1 Paediatric Clinic, Clinical Centre University of Sarajevo, Sarajevo, Bosnia and Herzegovina; 2 Health Centre of Canton Sarajevo, Sarajevo, Bosnia and Herzegovina; 3 Faculty of Medicine, University of Sarajevo, Sarajevo, Bosnia and Herzegovina; 4 Institute for Transfusion Medicine of the Federation of Bosnia and Herzegovina, Sarajevo, Bosnia and Herzegovina; 5 Clinical Immunology, Clinical Center University of Sarajevo, Sarajevo, Bosnia and Herzegovina

**Keywords:** Juvenile idiopathic arthritis, HLA class II alleles, treatment response, disease activity, prognostic marker

## Abstract

Juvenile idiopathic arthritis (JIA) is the most common chronic inflammatory autoimmune disease in childhood, significantly contributing to both short- and long-term disability. While certain human leukocyte antigen (HLA) class II alleles are known to be associated with specific subgroups of JIA, emerging evidence suggests a strong correlation between these alleles and treatment response. This study involved 143 JIA patients diagnosed according to International League of Associations for Rheumatology criteria. Each patient underwent HLA class II typing, including HLA-B27, as well as tests for rheumatoid factor (RF) and antinuclear antibodies (ANA). Comprehensive rheumatological assessments were conducted at diagnosis, with follow-ups at three and six months post-onset. After six months of methotrexate (MTX) treatment, patients were categorized as responders or non-responders. Responders achieved clinically inactive disease based on the American College of Rheumatology Provisional Criteria for Defining Clinical Inactive Disease and Clinical Remission. Non-responders, who did not reach clinically inactive disease after six months of treatment, required the addition of another non-biological disease-modifying antirheumatic drug (DMARD) or a biological DMARD. Our analysis revealed that the HLA-DRB1*01 allele is a significant prognostic marker for therapeutic response, predicting therapeutic resistance (*P* ═ 0.01). The most prevalent HLA-DRB1 alleles in the treatment-resistant group were HLA-DRB1*08:11 (11.3%), HLA-DRB1*01:01 (8.5%), HLA-DRB1*01:13, HLA-DRB1*04:11 (7%), HLA-DRB1*08:13, and HLA-DRB1*08:15 (4.2%). These findings highlight the critical role of HLA class II alleles in pediatric rheumatology, particularly in relation to treatment response and disease prognosis. In the era of personalized medicine, understanding the genetic contributions to treatment response and outcomes in JIA patients is essential. A key limitation of this study was the lack of comparison of treatment responses across different JIA subtypes. Future studies should prioritize evaluating MTX efficacy within specific JIA subgroups to enable a more tailored understanding of its effectiveness.

## Introduction

Juvenile idiopathic arthritis (JIA) is the most common chronic immune-mediated disease in children and a major cause of disability. In the past 20 years, advancements in understanding its pathogenesis have revolutionized treatment, reducing morbidity and improving quality of life.

The exact cause of JIA is unknown, but studies on twins and relatives suggest a genetic predisposition. Human leukocyte antigen (HLA) class II alleles are linked to specific JIA subgroups [1]. In clinical practice, identifying genetic markers associated with poor prognosis and therapy resistance is crucial, as patients with these markers require early, aggressive treatment, including biologic drugs.

Of all the genetic loci associated with JIA, the HLA locus has the most significant impact on genetic susceptibility, largely because of its key role in driving autoimmune destruction [2]. HLA-DRB1*08 is particularly implicated in JIA predisposition, especially in oligoarticular (oJIA) forms rather than polyarticular forms (pJIA). HLA-DRB1*01 and HLA-DRB1*04 are associated with rheumatoid factor (RF)-positive JIA, while HLA-DRB1*11 is a risk factor for oJIA, and HLA-DRB1*04 is linked to systemic JIA [3]. The goal of modern JIA treatment is complete remission, not merely symptom relief. Achieving remission within a critical therapeutic window is essential for a favorable outcome, and early aggressive therapy improves treatment response [4]. While clinical and laboratory risk factors for severe JIA are well studied, there is a lack of research in Bosnia and Herzegovina on the impact of genetic factors on prognosis and therapy response. Studies on HLA class II alleles could lead to improved, biologically guided disease classification [2]. The degree of response to immunomodulatory therapy in patients with JIA and rheumatoid arthritis (RA) is determined by genes located within the HLA class II region [5, 6]. Detecting HLA class II alleles in JIA patients that predict poor therapeutic response and prognosis could enable the development of personalized treatment protocols from the time of diagnosis. This study aims to assess the association and prognostic significance of HLA class II alleles with treatment response in JIA, contributing to personalized care in pediatric rheumatology.

## Materials and methods

### Study design and participants

This combined retrospective–prospective cohort study was conducted on 143 pediatric patients with JIA treated at the Pediatric Clinic of the Clinical Center of the University of Sarajevo, Department of Allergology, Rheumatology and Clinical Immunology from April 2019 to March 2023. The patient cohort represents a diverse geographic population from across the Federation of Bosnia and Herzegovina. Patients were included in the study if they had been diagnosed with JIA, were at least 12 months old or younger than 18 years old, received six months course of methotrexate (MTX) in addition to non-steroidal anti-inflammatory drugs, a single dose of intra-articular steroids per joint and systemic corticosteroids, and were admitted to the Pediatric clinic of the Clinical Center of the University of Sarajevo. The diagnosis of JIA was established by pediatric rheumatologist and immunologist according to the criteria of International League Against Rheumatism (ILAR), which defines the disease as arthritis lasting longer than six weeks in patients younger than 16 years, after excluding all other known causes of arthritis, such as infections, malignancies, or other immune-mediated diseases [7]. Patients with systemic JIA were excluded in case of significant systemic inflammatory characteristics of the disease and the differing treatment protocols, which required a prompt initiation of biologic therapies [8].

Uveitis was diagnosed by a clinician ophthalmologist, specializing in a pediatric ophthalmology, using a slit lamp and biomicroscope.

### Treatment

The study inclusion criteria required that, during the first six months following diagnosis, patients were treated exclusively with MTX alongside non-steroidal anti-inflammatory drugs, a single intra-articular steroid dose per joint, and systemic corticosteroids. Systemic corticosteroids were limited to a dose of 0.5 mg/kg of body weight and administered only during the first two months of MTX treatment. This dosage of systemic corticosteroids (prednisone) forms part of our “bridging” therapy approach, which is employed in our department to induce rapid remission while awaiting the delayed onset of MTX’s effects. This approach is particularly important in settings where initiating biological therapy at the start of treatment for certain cases of JIA may not always be feasible, despite its known benefits.

### Outcomes and evaluation

Patients were evaluated three months after initiating therapy, and treatment response was assessed after six months. Patients were classified as responders or non-responders. Responders were defined as those achieving clinically inactive disease according to the American College of Rheumatology Provisional Criteria for Defining Clinical Inactive Disease and Clinical Remission (also known as the Wallace Criteria). Clinically inactive disease was defined by five criteria: no joints with active arthritis; absence of fever, rash, serositis, splenomegaly, or generalized lymphadenopathy attributable to JIA; no active uveitis; normal erythrocyte sedimentation rate (ESR) or C-reactive protein (CRP) (if both were tested, both had to be normal); and a physician global assessment (PhGA) indicating no disease activity (the best score on the scale used, typically 0/10), with morning stiffness <15 min [9]. Non-responders were defined as those who did not achieve clinically inactive disease after six months of treatment, necessitating the addition of another non-biological disease-modifying antirheumatic drug (DMARD) or a biological DMARD. The primary outcome of this study was the identification of a prognostic factor for DMARD therapy. Secondary outcomes included the association of other laboratory tests (e.g., antinuclear antibodies [ANA] and RF) with therapy outcomes and JIA-associated conditions, such as uveitis.

### Laboratory analyses

Serum levels of RF were measured using turbidimetry, and ANA titer was determined using indirect immunofluorescence. All patients underwent HLA-B27 and HLA class II typing using peripheral blood samples at the Institute for Transfusion Medicine of the Federation of Bosnia and Herzegovina in Sarajevo, Bosnia and Herzegovina. HLA-B27 was determined using the PCR-SSO method with the OLERUP HLA-B27 kit, following the manufacturer’s instructions, which included a 96 ^∘^C denaturation step for 5 min, followed by annealing and extension steps specific to the assay. For HLA class II typing, HLA-DQ was determined using the “Innotrain SSP HLA DQ low resolution” kit, while HLA-DRB1 was assessed with “IMMUNOCOR LIFECO+DES SSO” and HLA-DQA1/B1 using “IMMUNOCOR Lifecodes”. All methods followed manufacturers’ guidelines and included quality control measures with positive and negative controls for each batch of samples tested. Confirmatory results underwent replicate testing, and ambiguous results were manually reviewed and retested as necessary. Finally, all assays were evaluated according to genotype-calling criteria.

### Ethical statement

This study was conducted in accordance with the principles outlined in the Helsinki Declaration. Ethical approval was granted by both the Ethics Committee of the Clinical Center of the University of Sarajevo and the Ethics Committee of the Faculty of Medicine, University of Sarajevo. Parents or guardians of all research participants signed an informed consent form prior to the collection of blood samples. All patient cases were coded to ensure anonymity, and data was managed within this encrypted system to protect patient identities.

### Statistical analysis

At the end of the study, statistical data processing was conducted. For statistical analysis, SPSS for Windows (version 19.0, SPSS Inc, Chicago, IL, USA) and Microsoft Excel (version 11, Microsoft Corporation, Redmond, WA, USA) were used. Nominal and ordinal variables were analyzed using the χ^2^ test. The symmetry of the distribution for continuous variables was assessed with the Kolmogorov–Smirnov test. When continuous variables deviated statistically significantly (*P* < 0.05) from a symmetric (Gaussian) distribution, the median and interquartile range were used to display the mean values and the measure of dispersion, and comparisons were made using non-parametric tests (Mann–Whitney *U* test). Otherwise, the independent *t*-test was applied, and the arithmetic mean and standard deviation were used to represent the average values.

For correlation between variables, Spearman’s rho correlation tests were conducted. The influence of individual variables on binary prediction (response to therapy), particularly the likelihood of a worse therapeutic outcome, was examined using univariate binary logistic regression. Variables that showed a statistically significant influence in univariate analysis were then included in a multivariate binary logistic regression. The reliability of the model was evaluated through a series of statistical tests, including the Hosmer and Lemeshow test, Cox and Snell R^2^, and Nagelkerke R^2^. A statistical significance threshold of α ═ 0.05 was established. Decisions regarding hypothesis acceptance or rejection were based on the *P* value from the statistical tests: if *P* ≥ α, the hypothesis was accepted; if *P* < α, the hypothesis was rejected. The results were thoroughly elaborated and documented, presented in absolute and relative numbers as well as statistical values, using statistical indicators, and organized into simple and understandable tables.

## Results

Of the 143 children included in the study, 56% were girls and 44% were boys. The average age of respondents was 9.2 ± 4.6 years, with the youngest being one year old and the oldest 16 years old. After six months of initial treatment with MTX, the subjects were divided into two groups based on their therapeutic response. The first group comprised children who reached inactive disease status according to the Wallace Criteria (*N* ═ 71), while the second group included non-responders (*N* ═ 72). Non-responders were defined as patients who did not achieve clinically inactive disease status after six months of treatment and subsequently required the addition of either another non-biological DMARD or a biological DMARD.

### Presence of RF, ANA and HLA-B27 in the sample

Five subjects (3%) in our sample tested positive for rheumatic factor (RF), while 48 subjects (33.6%) had a positive antinuclear antibody (ANA). Positive HLA-B27 was detected in 27 subjects (18.9%).

### Predictive influence of examined variables on therapeutic response

Using univariate regression analysis, we examined whether and to what extent the presence of HLA-DRB1*01 has predictive significance on the outcome of immunomodulatory therapy after six months of treatment, specifically on the appearance of therapeutic resistance to first-line of treatment. HLA-DRB1*01 was found to be a statistically significant predictor of therapy outcome (*P* ═ 0.01) and (Table 1 and Table 2). Subjects in whom HLA-DRB1*01 was detected had a three times higher likelihood of not responding to the MTX (odds ratio [OR] ═ 2.97) in our sample. In the population, this likelihood ranges from 1.3 to 6.8 times.

**Table 1:** Baseline and demographic data of study participants

**Variable**	**Value**
**Participants, *n* (%)**	143 (100.0)
*Males, n (%)*	63 (44)
Responders, *n*	27
Non-responders, *n*	36
*Females, n (%)*	80 (56)
Responders, *n*	44
Non-responders, *n*	36
**Age, average (range), *y***	9.2 (1–16)
*Males*	8.6 (2–15)
Responders	9.7 (3–15)
Non-responders	7.8 (2–15)
*Females*	9.66 (1–16)
Responders	8.4 (1–16)
Non-responders	11.2 (3–16)
*Study cohorts*	
**Responders group, *n* (%)**	72 (50.3)
Oligoarticular type	56 (77.8)
Polyarticular type, RF negative	9 (12.5)
ERA	7 (9.7)
Psoriatic arthritis	0 (0)
**Non-responders group, *n* (%)**	71 (49.7)
Oligoarticular type	9 (12.7)
Polyarticular type, RF positive	7 (9.9)
Polyarticular type, RF negative	35 (49.3)
ERA	17 (23.9)
Psoriatic arthritis	3 (4.2)
*Laboratory findings*	
**ESR**	
Responders, median [IQR]	12.5 [6.0–15.75]
Non-responders, median [IQR]	28.0 [11.0–42.0]
**C-reactive protein**	
Responders, median [IQR]	5.0 [3.0–20.0]
Non-responders, median [IQR]	6.2 [3.0–20.0]
**Rheumatoid factor, *n* (%)**	5 (2.1)
Responders, *n*	0
Non-responders, *n*	5
**ANA, *n* (%)**	48 (33.6)
Responders, *n*	35
Non-responders, *n*	13
**HLA-B27, *n* (%)**	27 (18.9)
Responders, *n*	18
Non-responders, *n*	9

n: Number; y: Years; RF: Rheumatoid factor; IQR: Interquartile range; ANA: Antinuclear antibody; HLA: Human leukocyte antigen; ESR: Erythrocyte sedimentation rate; ERA: Enthesitis-related arthritis.

**Table 2:** Prognostic influence of the presence of HLA-DRB1*01 on therapeutic response

**Variable**	**Regression coefficient**	**S.E.**	**Wald**	**Regression coefficient exponential**	**95% CI**	***P* value**
HLA-DRB1*01 Presence	1.089	0.425	6.570	2.971	1.292–6.831	0.010
Constant	−0.256	0.192	1.772	0.774	–	0.183

HLA-DRB: Human leukocyte antigen DRB; S.E.: Standard error; CI: Confidence interval.

### HLA class II alleles associated with therapeutically resistant JIA

In another group of patients with JIA, who did not achieve low disease activity after six months of treatment and required the introduction of an additional non-biological or biological disease-modifying drug, we identified other HLA class II alleles. Table 3 lists the HLA-DRB1 alleles of all subjects with JIA resistant to the MTX. Besides examining the influence of HLA-DRB1*01 on the therapeutic resistance of patients with JIA, we aimed to determine the degree of association of other HLA class II alleles with a disease that requires the rapid introduction of second-line therapeutic drugs. In the second group of therapeutically resistant subjects, the most frequently identified alleles from the HLA-DRB1 group were: HLA-DRB1*08:11 (11.3%), HLA-DRB1*01:01 (8.5%), HLA-DRB1*01:13 and HLA-DRB1*04:11 (7%), HLA-DRB1*08:13, HLA-DRB1*08:15 (4.2%) (Table 3).

**Table 3:** HLA-DRB1 in therapeutically resistant patients with JIA (non-responders)

**HLA-DRB1 allele type**	***n***	**Percentage**
HLA-DRB1*01	23	32.4
*Homozygote*		
HLA-DRB1*01:01	6	8.5
*Heterozygotes*		
HLA-DRB1*01:03	2	2.8
HLA-DRB1*01:08	2	2.8
HLA-DRB1*01:11	2	2.8
HLA-DRB1*01:12	2	2.8
HLA-DRB1*01:13	5	7.0
HLA-DRB1*01:15	2	2.8
HLA-DRB1*01:16	2	2.8
HLA-DRB1*03	5	7.0
*Heterozygotes*		
HLA-DRB1*03:07	1	1.4
HLA-DRB1*03:08	2	2.8
HLA-DRB1*03:13	1	1.4
HLA-DRB1*03:16	1	1.4
HLA-DRB1*04	11	15.5
*Homozygote*		
HLA-DRB1*04:04	1	1.4
*Heterozygotes*		
HLA-DRB1*04:11	5	7.0
HLA-DRB1*04:14	2	2.8
HLA-DRB1*04:15	2	2.8
HLA-DRB1*04:16	1	1.4
HLA-DRB1*07	4	5.6
*Homozygote*		
HLA-DRB1*07:07	1	1.4
*Heterozygotes*		
HLA-DRB1*07:11	1	1.4
HLA-DRB1*07:12	1	1.4
HLA-DRB1*07:16	1	1.4
HLA-DRB1*08	15	21.1
*Heterozygotes*		
HLA-DRB1*08:11	8	11.3
HLA-DRB1*08:13	3	4.2
HLA-DRB1*08:14	1	1.4
HLA-DRB1*08:15	3	4.2
HLA-DRB1*11	7	9.9
*Heterozygotes*		
HLA-DRB1*11:12	1	1.4
HLA-DRB1*11:13	2	2.8
HLA-DRB1*11:14	2	2.8
HLA-DRB1*11:15	1	1.4
HLA-DRB1*11:16	1	1.4
HLA-DRB1*13	4	5.6
*Homozygote*		
HLA-DRB1*13:13	1	1.4
*Heterozygotes*		
HLA-DRB1*13:14	1	1.4
HLA-DRB1*13:15	1	1.4
HLA-DRB1*13:16	1	1.4
HLA-DRB1*14	1	1.4
*Homozygote*		
HLA-DRB1*14:14	1	1.4
HLA-DRB1*15	1	1.4
*Homozygote*		
HLA-DRB1*15:15	1	1.4
**Total**	71	100.00%

HLA-DRB: Human leukocyte antigen DRB; JIA: Juvenile idiopathic arthritis.

The most prevalent HLA-DQA1 alleles in our sample were: HLA-DQA1*01:05 (22.9%), HLA-DQA1*01:01 (22.5%), and HLA-DQA1*01:04. (12.7%) (Table 4).

**Table 4:** HLA-DQA1(a) therapeutically resistant patients with JIA (non-responders)

**HLA-DQA1 allele type**	***n***	**Percentage**
HLA-DQA1*01	49	69.0
*Homozygote*		
HLA-DQA*01:01	16	22.5
*Heterozygotes*		
HLA-DQA*01:03	7	9.9
HLA-DQA*01:04	9	12.7
HLA-DQA*01:05	17	23.9
HLA-DQA1*02	4	5.63
*Homozygote*		
HLA-DQA*02:02	1	1.4
*Heterozygote*		
HLA-DQA*02:05	3	4.2
HLA-DQA1*03	8	11.27
*Homozygote*		
HLA-DQA1*03:03	1	1.4
*Heterozygote*		
HLA-DQA1*03:05	7	9.9
HLA-DQA1*04	7	9.9
*Heterozygote*		
HLA-DQA1*04:05	7	9.9
HLA-DQA1*05	3	4.2
*Homozygote*		
HLA-DQA1*05:05	3	4.2
**Total**	71	100.0

HLA-DQA: Human leukocyte antigen DQA; JIA: Juvenile idiopathic arthritis.

For HLA-DQB1 the most prevalent alleles were– HLA-DQB1*03:05 (14.1%) and HLA-DQB1*05:05 and HLA-DQB1*05:06 (11.3%) (Table 5).

**Table 5:** HLA-DQB1 in patients with therapeutically resistant JIA (non-responders)

**HLA-DQB1(a)** **allele type**	***n***	**Percentage**
HLA-DQB1*01	6	8.5
*Homozygote*		
HLA-DQB*01:01	4	5.6
*Heterozygotes*		
HLA-DQB*01:03	2	2.8
HLA-DQB1*02	12	16.9
*Homozygote*		
HLA-DQB1*02:02	1	1.4
*Heterozygote*		
HLA-DQB1*02:03	4	5.6
HLA-DQB1*02:04	2	2.8
HLA-DQB1*02:05	4	5.6
HLA-DQB1*02:06	1	1.4
HLA-DQB1*03	25	35.2
*Homozygote*		
HLA-DQB1*03:03	7	9.9
*Heterozygotes*		
HLA-DQB1*03:01	1	1.4
HLA-DQB1*03:04	4	5.6
HLA-DQB1*03:05	10	14.1
HLA-DQB1*03:06	3	4.2
HLA-DQB1*04	8	11.3
*Heterozygotes*		
HLA-DQB1*04:05	4	5.6
HLA-DQB1*04:06	4	5.6
HLA-DQB1*05	16	22.5
*Homozygote*		
HLA-DQB1*05:05	8	11.3
*Heterozygote*		
HLA-DQB1*05:06	8	11.3
HLA-DQB1*13	1	1.4
*Heterozygote*		
HLA-DQB1*13:06	1	1.4
**Total**	71	100.0

HLA-DQB: Human leukocyte antigen DQB; JIA: Juvenile idiopathic arthritis.

### List of HLA-DRB1 class II alleles present in patients with JIA-associated uveitis

Five subjects with JIA were diagnosed with uveitis. Among them, two subjects had HLA-DRB1*08:01, while one subject each had HLA-DRB1*01:02; HLA-DRB1*04:11; HLA-DRB1*11:14 (Table 6).

**Table 6:** HLA-DRB1 in relation to diagnosed uveitis

**HLA-DRB1 allele type**	**Uveitis**		
	**Present**	**Absent**	**Total**
HLA-DRB1*01			
*Homozygote*			
HLA-DRB1*01:01	6	0	6
*Heterozygotes*			
HLA-DRB1*01:03	2	1	3
HLA-DRB1*01:04	1	0	1
HLA-DRB1*01:07	1	0	1
HLA-DRB1*01:08	3	0	3
HLA-DRB1*01:11	3	0	3
HLA-DRB1*01:12	3	0	3
HLA-DRB1*01:13	8	0	8
HLA-DRB1*01:15	3	0	3
HLA-DRB1*01:16	2	0	2
HLA-DRB1*02			
*Heterozygote*			
HLA-DRB1*02:14	1	0	1
HLA-DRB1*03			
*Heterozygotes*			
HLA-DRB1*03:04	1	0	1
HLA-DRB1*03:07	3	0	3
HLA-DRB1*03:08	2	0	2
HLA-DRB1*03:11	2	0	2
HLA-DRB1*03:12	1	0	1
HLA-DRB1*03:13	4	0	4
HLA-DRB1*03:14	1	0	1
HLA-DRB1*03:16	2	0	2
HLA-DRB1*04			
*Homozygote*			
HLA-DRB1*04:04	2	0	2
*Heterozygotes*			
HLA-DRB1*04:05	1	0	1
HLA-DRB1*04:11	4	1	5
HLA-DRB1*04:12	1	0	1
HLA-DRB1*04:13	2	0	2
HLA-DRB1*04:14	3	0	3
HLA-DRB1*04:15	4	0	4
HLA-DRB1*04:16	7	0	7
HLA-DRB1*07			
*Homozygote*			
HLA-DRB1*07:07	1	0	1
*Heterozygotes*			
HLA-DRB1*07:11	3	0	3
HLA-DRB1*07:12	2	0	2
HLA-DRB1*07:13	3	0	3
HLA-DRB1*07:15	1	0	1
HLA-DRB1*07:16	1	0	1
HLA-DRB1*08			
*Heterozygotes*			
HLA-DRB1*08:11	6	2	8
HLA-DRB1*08:13	3	0	3
HLA-DRB1*08:14	2	0	2
HLA-DRB1*08:15	3	0	3
HLA-DRB1*10			
*Heterozygotes*			
HLA-DRB1*10:11	1	0	1
HLA-DRB1*11			
*Homozygote*			
HLA-DRB1*11:11	1	0	1
*Heterozygotes*			
HLA-DRB1*11:12	1	0	1
HLA-DRB1*11:13	6	0	6
HLA-DRB1*11:14	3	1	4
HLA-DRB1*11:15	6	0	6
HLA-DRB1*11:16	1	0	1
HLA-DRB1*12			
*Heterozygotes*			
HLA-DRB1*12:15	1	0	1
HLA-DRB1*13			
*Homozygote*			
HLA-DRB1*13:13	2	0	2
*Heterozygotes*			
HLA-DRB1*13:14	1	0	1
HLA-DRB1*13:15	3	0	3
HLA-DRB1*13:16	4	0	4
HLA-DRB1*14			
*Homozygote*			
HLA-DRB1*14:14	1	0	1
HLA-DRB1*15			
*Homozygote*			
HLA-DRB1*15:15	5	0	5
*Heterozygotes*			
HLA-DRB1*15:13	1	0	1
HLA-DRB1*15:16	1	0	1
HLA-DRB1*16			
*Homozygote*			
HLA-DRB1*16:16	2	0	2
**Total**	138	5	143

HLA-DRB: Human leukocyte antigen DRB.

### Association between ANA and JIA-associated uveitis

ANA were positive in 48 subjects with JIA, including two patients (4.2%) with uveitis (Table 7). Among the three cases of uveitis where ANA was negative, the prevalence was 3.2%.

**Table 7:** Relation between uveitis and the presence of ANA in patients with JIA

	**Uveitis**		
	**Present**	**Absent**	**Total**
ANA positive	2 (4.2)	46 (95.8)	48 (100.0)
ANA negative	3 (3.2)	92 (96.8)	95 (100.0)
Total	5 (3.5)	138 (96.5)	143 (100.0)

Data are presented as number (percentage). ANA: Antinuclear antibody test; JIA: Juvenile idiopathic arthritis.

### Correlation of the presence of HLA-B 27 with therapeutically resistant forms of JIA

In the total sample of 143 patients, HLA-B27 was detected in 27 subjects, of whom 18 (66.7%) exhibited high or moderate disease activity after six months of therapy, indicating therapeutic resistance. Among the 116 HLA-B27-negative subjects, 53 (45.7%) displayed high or moderate disease activity after six months. The difference in therapeutic resistance between HLA-B27-positive and -negative patients approached statistical significance (*P* ═ 0.05) (Table 8).

**Table 8:** Correlation of HLA-B27 and therapeutically resistant forms of JIA

	**Treatment outcome**		
	**Responders**	**Non-responders**	**Total**
HLA-B27 positive	9 (33.3)	18 (66.7)	27 (100.0)
HLA-B27 negative	63 (54.3)	53 (45.7)	116 (100.0)
Total	72 (50.3)	71 (49.7)	143 (100.0)

Data are presented as number (percentage). HLA-B27: Human leukocyte antigen B27; JIA: Juvenile idiopathic arthritis.

## Discussion

The goal of modern rheumatology is to treat diseases promptly and effectively to prevent the progression of inflammation and irreversible damage to joint structures. Making strategic decisions about treatment and drug selection is crucial for the successful management of JIA. Effective JIA treatment relies on accurately assessing disease severity, the potential for achieving remission, therapeutic resistance, and the likelihood of relapse [10].

Our research aimed to explore the predictive power of HLA class II alleles on the development of therapeutically resistant JIA. The study included 143 patients, with 56% girls and 44% boys, and an average age of 9.2 ± 4.6 years. The age range was from one to 16 years. There was no statistically significant difference in the age at diagnosis between male and female subjects (*P* ═ 0.191).

Testing for RF, ANA, and HLA-B27 was conducted, and HLA typing was performed. ANA was detected in 48 subjects (33%). Although the ANA test is not used for diagnosis, it is an important prognostic marker for the development of uveitis. The overall seroprevalence of ANA positivity in JIA patients is less than 50% [11]; however, patients with a positive ANA test are at higher risk of developing uveitis and should be closely monitored.

While positive ANA is more common in children with JIA compared to the healthy population, its presence does not increase the likelihood of developing JIA. ANA can often be falsely or transiently positive following an infection [12].

The percentage of HLA-B27 positivity in our cohort was 27%, consistent with data from a Polish study [6] and higher than the prevalence reported by Murray et al. [13] for patients in the United States (14%) and by Thomson et al. [14] in a cohort from Great Britain (16.9%). This difference may be explained by the genetic variability of the study populations, who come from different ethnic backgrounds. In healthy individuals, the frequency of HLA-B27 occurrence is 9.6% [15]. The pathophysiology of HLA-B27-related diseases, including JIA, is thought to involve several mechanisms. HLA-B27 may present microbial peptides that resemble self-peptides, leading to cross-reactivity and autoimmunity. Additionally, HLA-B27 may alter the repertoire of peptides presented to T cells, potentially triggering an inappropriate immune response and resulting in inflammation. HLA-B27 positivity may also interact with the gut microbiota, promoting immune dysregulation and systemic inflammation—a mechanism believed to contribute to spondyloarthropathies and other HLA-B27-associated conditions [16].

Given these findings, it is particularly important in both clinical practice and public health contexts to assess whether genetic and other biologically determined markers in pediatric JIA patients in Bosnia and Herzegovina are associated with poor prognosis or resistance to standard immunomodulatory therapy. Identifying such markers could guide the early initiation of aggressive therapies, including biological drugs, since the goal of modern JIA treatment is to achieve complete disease remission, not merely symptom relief. In modern rheumatology, ensuring patients achieve disease inactivity and remission through appropriate initial therapeutic strategies a top priority.

Data from multiple registries emphasize the importance of the “window of opportunity” for early treatment with DMARDs. Initiating DMARD therapy, especially within the first six months, has been shown to improve functional outcomes, reduce disease activity, and decrease the need for corticosteroids. This highlights the importance of early aggressive therapy upon diagnosis [17, 18]. However, the use of aggressive drugs in pediatric patients can lead to significant side effects, such as hepatotoxicity, bone marrow suppression, and an increased risk of malignancies—particularly lymphoma, as reported by Diak et al. [19] (2010), who found an incidence of lymphoma four times higher in children treated with TNF inhibitors. Additionally, biological therapies are associated with high treatment costs. These factors underscore the need to identify biological markers for individual JIA patients to guide informed therapy decisions.

Over the past 30 years, numerous studies have demonstrated a positive correlation between HLA class II alleles and JIA. Associations have been identified between HLA-DRB1*08, HLA-DRB1*11, HLA-DRB1*13, and HLA-DRB1*01 and the presence of inflammatory, immune-mediated arthritis in children [20]. In a cohort of Japanese patients, HLA-DRB1*04 was linked to polyarticular JIA, while HLA-DRB1*02 was associated with more complex forms of JIA-associated uveitis [21]. Norwegian and Polish studies have also shown a notable prevalence of HLA-DRB1*08 among JIA patients [22]. Additionally, a genome-wide association study by Haasnoot et al. found that a female predisposition to JIA-associated uveitis was related to the tyrosine–serine–threonine (YST) motif in the beta-1 subunit of the HLA-DR receptor. Modifications to the HLA-DRB1 receptor may influence autoantigen presentation, immune cell activation, and immune tolerance, contributing to inflammation [23].

Studies on adult patients with RA also indicate geographic differences in HLA-DRB risk alleles. In Sudanese populations, HLA-DRB1*04 and HLA-DRB1*10 are linked to an increased risk of RA [24]. Among RA patients, those with the HLA-DRB1*04 allele tend to respond better to abatacept therapy compared to those without this allele [25]. In children with a polyarticular course of JIA, HLA-DRB1*08.01 and DRB1*11.03 alleles are associated with an earlier disease onset—3.9 years on average, compared to 7.3 years for those without these alleles [26]. While various factors influence HLA-DRB1 prevalence, it is also notable that HLA-DRB1 has been associated with therapy resistance [27]. Although the pathophysiology remains largely elusive, it is clear from both basic and translational studies that environmental, genetic, and epigenetic factors contribute to disease development [28].

In our research, we examined the predictive influence of HLA-DRB1*01 on the development of therapeutic resistance to “first-line” immunomodulatory therapy (non-steroidal anti-inflammatory drugs, a single dose of intra-articular steroids per affected joint, and systemic corticosteroids limited to 0.5 mg/kg of body weight during the first two months of MTX therapy). In our patient cohort, we demonstrated that HLA-DRB1*01 is a statistically significant predictor of therapeutic resistance (*P* ═ 0.01). Subjects carrying this allele have a three times greater likelihood of non-response to first-line therapy (OR ═ 2.97). HLA-DRB1*01 belongs to the family of shared epitope (SE). The hypothesis suggests that the presence of SE sequences enables the presentation of self-antigens to T lymphocytes, which is pivotal in the development of RA [29]. The role of SE sequences in immunology is likely tied to their effects on adaptive immunity. Those who carry the SE exhibit elevated HLA-DR expression on B cells, which interact with T cell receptors. This interaction leads to elevated CXCR4 expression on memory CD4+ T cells [30]. SE alleles are also associated with heightened disease activity in RA and a reduced likelihood of achieving DMARD-free remission. Additionally, they can predict a suboptimal response to conventional synthetic DMARDs (csDMARDs), particularly MTX [31]. Recent studies suggest that specific HLA-DRB1 variants may predict unfavorable disease progression, including an increased risk of radiographic damage and a higher incidence of interstitial lung disease and lymphoproliferative disorders. Identifying high-risk patients carrying the HLA-DRB1 risk allele could help personalize therapeutic approaches, as early aggressive immunosuppressive treatment has been shown to provide significant clinical benefits for these patients [32].

Identifying the HLA-DRB1 allele at the time of JIA diagnosis could assist rheumatologists in developing more effective treatment protocols. Patients with the HLA-DRB1*01 allele should be closely monitored for disease activity, as they are more likely to exhibit resistance to initial therapy. Early aggressive immunomodulatory treatments can induce may lead to faster disease remission, prevent joint damage, and reduce disability. Our findings align with a comprehensive meta-analysis by De Silvestri et al., which reviewed medical databases and publications on the association between HLA class II alleles and JIA in children and adolescents up to age 18. Their meta-analysis, like our study, established a statistical association between HLA-DRB1*01 and polyarticular, therapy-resistant JIA. They also identified HLA-DRB1*04 as a predictor of RF-positive JIA with therapeutic resistance [3].

Hersh and Prahalad [33] demonstrated the predictive power of HLA-DRB1*01 for the development of more severe and treatment-resistant forms of JIA. A comprehensive study by Hollenbach et al., which included HLA class II typing results from 820 JIA patients, found that HLA-DRB1*08 is associated with the polyarticular course of JIA in children over six, while HLA-DRB1*11 is linked to the polyarticular form in younger patients and the oligoarticular form. Given the variability in genetic analyses across different geographic and national contexts, it is unsurprising that studies from various parts of the world report differing results [26].

Despite differences in nomenclature, adult and pediatric seropositive RA share many characteristics, including the types of joints involved, the formation of rheumatoid nodules, disease progression, and responses to therapy. The RF-positive polyarticular form of JIA shows an increased incidence of HLA-DRB1*01 compared to the healthy population. This form of JIA is noted in the literature for its higher resistance to therapy. Both adult and pediatric seropositive arthritis share not only HLA associations but also non-HLA loci, facilitating comparisons between these patient groups [22, 23].

In addition to finding that HLA-DRB1*01 was a statistically significant predictor of therapeutically resistant disease in our sample, and considering the established role of HLA class II associated genes in the etiopathogenesis of JIA [3], our study aimed to explore how other HLA class II alleles are associated with disease forms requiring the rapid introduction of second-line therapies. Among the second group of therapeutically resistant subjects, the most frequently represented alleles in the HLA-DRB1 group were: HLA-DRB1*08.11 (11.3%), HLA-DRB1*01.01 (8.5%), HLA-DRB1*01.13 and HLA-DRB1*04.11 (7%), HLA-DRB1*08.03 and HLA-DRB1*08.15 (4.2%).

Our findings align with the conclusions of a large meta-analysis by De Silvestri et al., which included studies on 2473 JIA patients and 9543 healthy pediatric controls. They demonstrated that the predisposing allele for JIA development, regardless of its type, is HLADRB1*08 (OR ≅ 6), and the most remarkable association was with oligo-articular (OR ═ 9.2, 95% CI ═ 6.7–13) and poly-articular JIA (OR ═ 6, 95% CI ═ 4.8–9) rather than with systemic JIA.

Furthermore, they indicated that HLA-DRB1*01 and HLA-DRB1*04 may contribute to genetic predisposition for RF-positive JIA. The meta-analysis also identified a significant number of subjects with HLA-DRB1*11, which we did not detect in our study. Genetic variability among ethnicities of respondents and our smaller sample size may account for this discrepancy [3].

The association of HLA-DRB1*01 with therapeutic resistance in JIA has further support from a study by Ali et al. [34] involving a Pakistani population, which found that patients with HLA-DRB1*03 were significantly more likely to have an inadequate response to MTX, a first-line immunomodulatory therapy. Similarly, O’Dell and colleagues demonstrated as early as 1998 that carriers of alleles, such as HLA-DRB1*04, HLA-DRB1*01, HLA-DRB1*10, and HLA-DRB1*14 in RA had a reduced likelihood of responding to first-line MTX therapy, specifically in achieving low disease activity (ACR 50). Patients with these alleles required the introduction of a combination therapy (MTX, sulfasalazine, and hydroxychloroquine) to achieve adequate disease control, in contrast to patients without predisposing alleles, who achieved remission with MTX (*P* < 0.0001). Published data clearly indicate an association between HLA class II alleles and therapeutic resistance in patients with immune-mediated arthritis [35].

The association between HLA class II alleles and JIA has been extensively studied, but several limitations and inconsistencies in the current literature must be acknowledged. Many studies on HLA alleles and JIA are retrospective, making them prone to biases, such as selection bias and recall bias. In contrast, prospective studies, which are less prone to these issues, are fewer in number. While the International League of Associations for Rheumatology (ILAR) criteria for JIA are widely used, not all studies adhere strictly to these guidelines, leading to potential misclassification of JIA subtypes. The majority of studies, including our own, have relatively small sample sizes, which limits the statistical power to detect significant associations between specific HLA alleles and therapeutic resistance.

Our cohort size consisted out of 143 patients who were balanced based on sex. However, this sample size could potentially limit the generalizability of our findings, particularly when it comes to detecting associations with less common alleles, considering that the JIA is a complex and heterogenous condition. Furthermore, this paper studies pediatric patients and the age limit set in the study was up to 18 years of age. That being said, obvious variations in patients’ physiology and pathophysiology due to age differences could influence the treatment outcomes. The reduced statistical power may hinder our ability to identify rare HLA alleles that could influence treatment resistance, leading to possible underestimation of their true impact.

Differences in predisposing HLA-DRB1 alleles across various ethnicities indicate the need for similar studies to explore genetic influences on therapeutic responses in patients with JIA and RA in Bosnia and Herzegovina. However, dissecting this study sample for subgroup analyses provided us with uneven data for different subgroups, which, we considered as insufficient for analysis and quite misleading. This is largely due to heterogenous sample, a well-established issue in this field [36–38]. Therefore, future studies in Bosnia and Herzegovina, as well as globally should aim to include larger sample sizes to better represent the population and increase the power to detect associations. Additionally, these studies should include longer follow-up periods to account for long-term therapeutic effects.

## Conclusion

In conclusion, our study marks a significant milestone as the first investigation in Bosnia and Herzegovina to examine the predictive role of HLA-DRB1*01 in developing therapeutic resistance in JIA. Our findings suggest that the HLA-DRB1*01 allele may help identify a subgroup of JIA patients likely to experience a more challenging therapeutic course. These results carry substantial clinical and practical implications, offering guidance for newly diagnosed patients and their families. Rheumatologists prescribing treatment need a reliable biological marker to assess the likelihood of a newly diagnosed patient developing a therapeutically resistant form of the disease. This information should be discussed thoroughly with parents, taking potential drug side effects into account, to collaboratively decide on the best therapeutic approach. Previous research has shown that having clearer expectations about disease progression can alleviate uncertainty and anxiety among parents facing a JIA diagnosis for their child, promoting better adherence to treatment protocols. One limitation of this study was the lack of comparative data on treatment responses across different JIA subtypes due to sample heterogeneity. Future research should prioritize evaluating therapeutic outcomes with MTX within each specific JIA subgroup to achieve a more tailored understanding of its effectiveness. Additionally, further studies are needed to validate these findings in larger cohorts and to explore the predictive value of other HLA alleles in therapeutic resistance.
